# Identification and Analysis of Dysfunctional Genes and Pathways in CD8^+^ T Cells of Non-Small Cell Lung Cancer Based on RNA Sequencing

**DOI:** 10.3389/fgene.2020.00352

**Published:** 2020-05-08

**Authors:** Xuefang Tao, Xiaotang Wu, Tao Huang, Deguang Mu

**Affiliations:** ^1^Affiliated Hospital of Shaoxing University, Shaoxing, China; ^2^Shanghai Engineering Research Center of Pharmaceutical Translation, Shanghai, China; ^3^Shanghai Institute of Nutrition and Health, Shanghai Institutes for Biological Sciences, Chinese Academy of Sciences, Shanghai, China; ^4^Department of Respiratory Medicine, Zhejiang Provincial People’s Hospital, People’s Hospital of Hangzhou Medical College, Hangzhou, China

**Keywords:** gene, CD8^+^ T cell, non-small cell lung cancer, RNA sequencing, feature selection, dysfunctional pathways

## Abstract

Lung cancer, the most common of malignant tumors, is typically of the non-small cell (NSCLC) type. T-cell-based immunotherapies are a promising and powerful approach to treating NSCLCs. To characterize the CD8^+^ T cells of non-small cell lung cancer, we re-analyzed the published RNA-Seq gene expression profiles of 36 CD8^+^ T cell isolated from tumor (TIL) samples and 32 adjacent uninvolved lung (NTIL) samples. With an advanced Monte Carlo method of feature selection, we identified the CD8^+^ TIL specific expression patterns. These patterns revealed the key dysfunctional genes and pathways in CD8^+^ TIL and shed light on the molecular mechanisms of immunity and use of immunotherapy.

## Introduction

Lung cancer, the most common of malignant tumors, is typically (∼80%) of the non-small cell (NSCLC) type (Zhan et al., 2017). Current therapies for NSCLC include surgery followed by adjuvant radiotherapy, chemoradiotherapy, and molecule-targeted therapy; these methods have produced excellent results (Antonicelli et al., 2013; Martinez et al., 2014; Nascimento et al., 2015). However, most patients with NSCLC are in the advanced or inoperable stage with limited treatment options, and the 5-year survival rate is still less than 20% (Siegel et al., 2012; van der Drift et al., 2012). Thus, innovative therapeutic approaches to achieve long-term disease control without obvious adverse reactions are needed.

Tumor-infiltrating lymphocytes are considered to play a critical role in the immune response to many human solid cancers. Most CD8^+^ T cells are cytotoxic T lymphocytes in the case of tumor-infiltrating lymphocytes (Farhood et al., 2019). In the immune response to cancer, these tumor-infiltrating CD8^+^ T cells have the potential to recognize specific antigens that are presented by the MHC class I receptor on cancer cells and target them for destruction. Studies have shown that immune infiltration by CD8^+^ cytotoxic T cells is significantly correlated with improved clinical outcome in non-small cell lung cancer (NSCLC) (Johnson et al., 2000; Welsh et al., 2005; Al-Shibli et al., 2008; Kawai et al., 2008). High density of tumor-infiltrating lymphocytes usually signified strong prognostic value (Hiraoka et al., 2006; Al-Shibli et al., 2008; Kawai et al., 2008; Schalper et al., 2015).

Currently, T-cell-based immunotherapies are a promising and innovative approach toward treating NSCLC. The development of anticancer drugs targeting T cells to enhance the immune response has shown great clinical benefit in NSCLC (Garon et al., 2015; Gettinger et al., 2015; Jia et al., 2015). Programmed death factor-1 (PD-1) expressed in tumor tissue plays a key role in downregulating T-cell activation and promoting tumor immune escape by binding to its ligand PD-L1, which is expressed on the surface of tumor cells (Pardoll, 2012; Dermani et al., 2019). Nivolumab, a PD-1 immune checkpoint inhibitor antibody, was recently approved by the United States Food and Drug Administration for treatment of patients with metastatic squamous NSCLC (Morgensztern and Herbst, 2016). It disrupts PD-1-mediated signaling and is linked to an anticancer immune response. Early clinical trials have indicated that PD-L1 expression on tumor-infiltrating lymphocytes and tumor cells may increase the response to PD-1-directed therapies in metastatic NSCLC (Herbst et al., 2014, 2016; Ramalingam et al., 2016). Therefore, tumor-infiltrating lymphocytes and the expression of PD-L1 are being considered as biomarkers capable of screening NSCLC patients most likely to respond to checkpoint antibody therapy (Johnson et al., 2014).

To identify the markers for CD8^+^ T cells in lung cancer, we compared the published RNA-Seq gene expression profiles of 36 CD8^+^ T cell isolated from tumor (TIL) samples and 32 adjacent uninvolved lung (NTIL) samples. With a Monte Carlo feature selection method, we identified the CD8^+^ TIL-specific expression patterns, which can accurately predict such cells. The original study of this published dataset identified 1,403 differentially expressed genes using DE-Seq with fold change greater than 1.5 and adjusted value of *p* < 0.05 (Ganesan et al., 2017). This number of genes is too numerous for use in a biomarker analysis along with the low expected utility of the set of statistically significant genes (Simon, 2008). Instead, we used a Monte Carlo feature selection method, which assembled a series of decision trees for classification of genes by importance (Draminski et al., 2008). The usefulness of this method has been evaluated by others (Li et al., 2019; Chen et al., 2020). The functional analysis of these genes and the CD8^+^ TIL signatures are presented in this study to help understand the molecular mechanisms of immunity and their possible relevance to immunotherapy.

## Materials and Methods

### The RNA-Seq Gene Expression Profiles of Non-Small Cell Lung Cancer

We downloaded the gene expression profiles of 36 CD8^+^ T cells isolated from tumor (TIL) samples and 32 adjacent uninvolved lung (NTIL) samples from the Gene Expression Omnibus (GEO) under accession number GSE90728 (Ganesan et al., 2017). All lung patients had non-small cell lung cancer (NSCLC). Other clinical details are available in Ganesan et al. (2017). The gene expression levels were quantified with HTSeq (Anders et al., 2015) after the RNA sequencing reads were mapped onto the human reference genome (hg19) using the TopHat software (Trapnell et al., 2009) by Ganesan et al. (2017). The processed matrix of 23,366 genes in 36 TIL samples and 32 NTIL samples was used to identify the key discriminative genes between TIL samples and 32 NTIL samples.

### The Monte Carlo Feature Selection Method

There have been many methods for identifying differentially expressed genes, such as the t-test, significance analysis of microarrays (SAM) (Tusher et al., 2001), and DESeq2 (Love et al., 2014). However, they typically only consider the statistical significance even though the statistically significant genes do not have discriminative ability (Simon, 2008). Since they do not consider the relationship between genes, they may be redundant or without known biological functions. To overcome these problems, we used a Monte Carlo feature selection method (Draminski et al., 2008; Cai et al., 2018; Chen et al., 2018a; Pan et al., 2018) to extract the CD8^+^ T-cell-specific gene expression patterns. The Monte Carlo feature selection method is powerful in discriminating features in a data set and has been widely used (Chen et al., 2018a, 2020; Chen L. et al., 2019; Chen X. et al., 2019; Li et al., 2019; Pan et al., 2019).

### The Monte Carlo Feature Selection Algorithm Works as Follows

Let us use *d* to denote the number of features, i.e., 23,366 genes in this study. To explain the feature selection algorithm, we used features instead of the expression level of genes since feature was a broader concept. The expression levels of genes can be features, but features can be any numerical vector.

First, *m* features (*m*≪*d*) are randomly selected for *s* times;

Then, *t* trees for each of the *s* subsets are constructed;

Last, *s*⋅*t* classification trees will be grouped to calculate a feature *g*’s relative importance (RI).

To be more specific, RI of feature *g* is based on how many times feature *g* is selected by the *s*⋅*t* trees and how much feature *g* contributes to the classification of the *s*⋅*t* trees. The equation of RI is

(1) $${\mathrm{RI}}_{g}=\sum_{\tau =1}^{s\,t}{\left(w\,A\,c\,c\right)}^{u}\,\sum_{n_{g}\,\left(\tau \right)}\text{IG}\,\left(n_{g}\,\left(\tau \right)\right)\,{\left(\frac{\mathrm{no}.\mathrm{in}\,n_{g}\,\left(\tau \right)}{\mathrm{no}.\mathrm{in}\,\tau }\right)}^{\nu }$$

in which *wAcc* is the weighted classification accuracy of decision tree τ, IG(*n*_*g*_(τ)) is the information gain of node *n*_*g*_(τ), which is a decision rule of feature *g*, (*no*.*in*n_g_(τ)) is the number of samples under node *n*_*g*_(τ), (*no*.*in*τ) is the number of samples in decision tree τ, and *u* and ν are additional tunable parameters, which adjust the influence of *wAcc* and $\frac{\mathrm{no}.\mathrm{in}\,n_{g}\,\left(\tau \right)}{\mathrm{no}.\mathrm{in}\,\tau }$, respectively (set to 1 by default).

The Monte Carlo feature selection method is a complex algorithm when the dataset is large. Therefore, a software called dmLab (Draminski et al., 2008), which can be downloaded from http://www.ipipan.eu/staff/m.draminski/mcfs.html was used to apply the Monte Carlo feature selection method.

After the RI values for all 23,366 genes were calculated, all these gene features were ranked as

(2) $$F=\left[f_{1},f_{2}\,\cdots \,f_{N}\right]$$

in which *N* is the total number of gene features, i.e., 23,366 in this study.

The gene features with smaller indices have greater RI value. In other words, the genes are sorted decreasingly. Since all the genes were ranked by importance, the top 500 genes are sufficient for identifying a potential biomarker for practical use. This set of genes was analyzed in the next step.

### The Support Vector Machine Classifier for CD8^+^ T Cells

Although all gene features may be ranked by their RI values (Monte Carlo feature selection), it was difficult to discern how many top features to select as optimal CD8^+^ T cell biomarkers.

To determine the number of features required for accurate classifier, we adopted an incremental feature selection (IFS) method (Wang S. et al., 2017; Zhang Y. H. et al., 2017; Chen et al., 2018b, c; Li et al., 2018a). First, 500 different feature sets *F*_1_,*F*_2_⋯*F*_500_ were constructed. In these feature sets, feature set *F*_*i*_ = [*f*_1_,*f*_2_⋯*f*_*i*_] included the top *i* features of *f* in Eq. (2). As explained above, features with a smaller index were more important, and features with a larger index were less important. These less important genes were more likely to introduce noise in the classifier and, therefore, decrease the performance of the classifier. Therefore, we needed to find the balance between signal (important features with small index) and noise (unimportant features with large index). For each feature set *F*_*i*_, a support vector machine (SVM) classifier was built based on these top *i* features, and their performance was evaluated with leave-one-out cross validation (LOOCV). An svm classifier can predict whether a cell was TIL based on its expression levels of the top *i* features/genes. Using the number of features as x-axis and their LOOCV accuracy as y-axis, an IFS curve can be plotted. The accuracy was the number of correctly predicted samples over the number of total samples. Based on the peak of IFS curve, the optimal number of gene features can be determined.

In this study, the SVM classifier was built using the R function svm from package e1017.^1^ The default parameters of R function svm were used to train the SVM models.

## Results

### The Relative Importance of Genes for CD8^+^ T Cells

As we described in the methods, the Monte Carlo feature selection method was adopted to analyze the gene expression profiles of 36 TIL samples and 32 NTIL samples. The goal was to identify the discriminative genes between TIL samples and NTIL samples. The 23,366 genes were ranked based on their relative importance calculated by the Monte Carlo feature selection algorithms.

The relative importance value reflected how well and how often this gene can be used to classify the TIL samples and NTIL samples in the resampling feature subsets on the decision trees. Since relative importance value integrates the information of many decision trees, it is a robust measurement that will not be easily influenced by noise. The genes can be ranked based on their relative importance values.

If a gene is important, it will rank at the top. All 23,366 genes were ranked, but only the top 500 genes were further analyzed for biomarker identification.

### The Key Genes and Pathways of CD8^+^ T Cells

**FIGURE 1:**
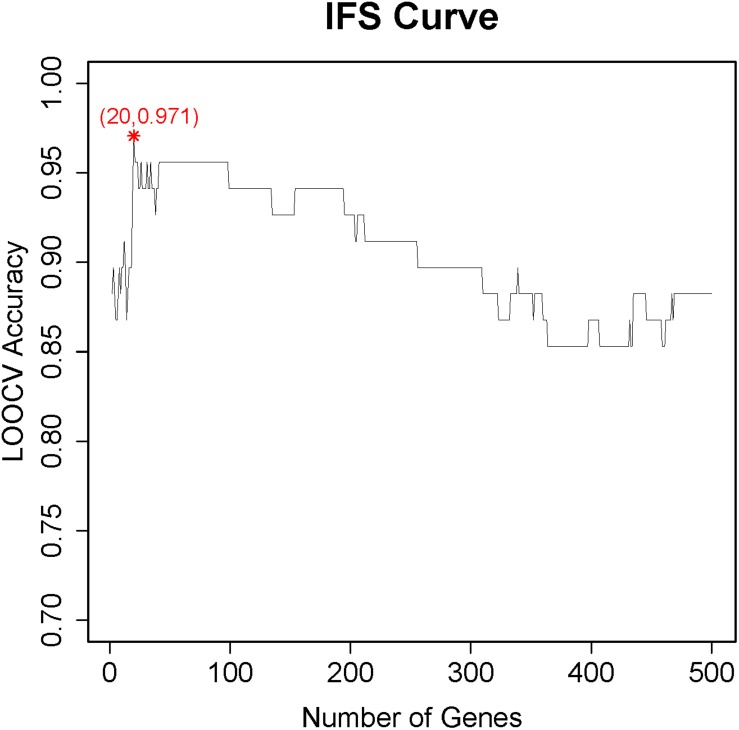
The incremental feature selection (IFS) curve to determine the optimal number of key genes for CD8^+^ T cells. The X-axis was the number of genes used to build the support vector machine (SVM) classifier, while the Y-axis was the prediction accuracy evaluated with leave-one-out cross validation (LOOCV). When the top 20 genes were used, the LOOCV accuracy was the highest, 0.971. These 20 genes were considered to be the key genes for CD8^+^ T cells.

After the genes were ranked based on their relative importance by the Monte Carlo feature selection, we applied the IFS method to further optimize the final key gene set that pertains to CD8^+^ T cells. We constructed 500 gene sets in which each gene sets included top *i* genes in the ranked gene list. Based on the number of genes and their prediction accuracy, we plotted the IFS curve in Figure 1. It can be seen that with the top 20 genes, the LOOCV accuracy was the highest, 0.971. Therefore, these 20 genes were considered as the key gene set of CD8^+^ T cells, and they are listed in Table 1. Even with the top two genes, SLCO3A1 and PXN, the accuracy was 0.882. Since there was no similar CD8^+^ T cell dataset, we searched these two genes against the CellMarker database (Zhang et al., 2018a). This curated database has 13,605 cell markers of 467 cell types among 158 human tissue types, and 9,148 cell markers of 389 cell types among 81 mouse tissue types. Based on the CellMarker database, SLCO3A1 was a marker for the natural killer T (NKT) cell; PXN was a cell marker for natural killer, CD4^+^ cytotoxic T cell, and effector CD8^+^ memory T (Tem) cell. All 20 genes were included in the 1,403 differentially expressed genes as identified by Ganesan et al. (2017).

**TABLE 1:** The 20 key genes for CD8+ T cells.

**Rank**	**Gene**	**Relative importance**
1	SLCO3A1	0.387
2	PXN	0.280
3	CKAP2	0.213
4	MGAT3	0.201
5	SFTPC	0.194
6	VCL	0.187
7	RASGRP2	0.182
8	PLAC8	0.170
9	AES	0.129
10	FAM65B	0.121
11	NHSL2	0.100
12	S100A10	0.099
13	RAB3GAP1	0.090
14	WIPF3	0.090
15	OSBPL5	0.089
16	CXCL13	0.089
17	GEM	0.085
18	S1PR1	0.083
19	TAGLN2	0.082
20	C16orf54	0.079

To test whether the classification model can affect the feature selection, we used the decision tree (R package rpart) instead of SVM, and the peak also appeared at 18, 19, and 20 with the highest LOOCV accuracy of 0.824. The 20 genes still performed the best. These results suggested the genes selected by IFS were robust to classifiers.

**FIGURE 2:**
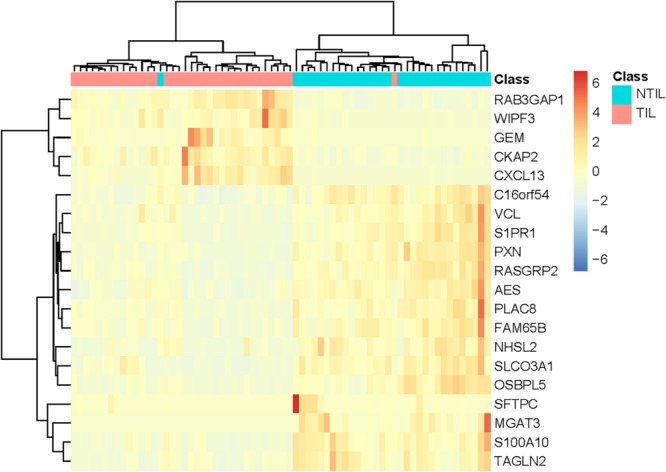
The heatmap of the 20 genes in TIL samples and NTIL samples. The sample classes were indicated on the first row: blue for NTIL samples and red for TIL samples. The two classes of samples were correctly clustered into two groups. Only two samples were not correctly clustered. It can be seen that RAB3GAP1, WIPF3, GEM, CKAP2, and C-X-C motif chemokine ligand 13 (CXCL13) were highly expressed in TIL samples, while other genes were lowly expressed in NTIL samples.

To explore the expression pattern of these 20 genes, we plotted the heatmap of these 20 genes and two classes of samples in Figure 2. It can be seen that the NTIL samples and TIL samples were correctly clustered into two groups. Only two samples were not correctly clustered. Within the 20 genes, RAB3GAP1, WIPF3, GEM, CKAP2, and C-X-C motif chemokine ligand 13 (CXCL13) were highly expressed in TIL samples, while the other genes were lowly expressed in NTIL samples.

To investigate the dysfunctional pathways affected by these genes, we did KEGG (Kyoto Encyclopedia of Genes and Genomes) enrichment analysis using WebGestalt (WEB-based GEneSeTAnaLysis Toolkit) (Wang J. et al., 2017) and found that hsa04062 chemokine signaling pathway was most significantly enriched with a *p* value of 6.12e-04 and odds ratio of 15.99.

## Discussion

### The Key Dysfunctional Genes in CD8^+^ T Cells

Biomarkers are of great significance for the diagnosis and treatment of cancer. Recent studies have found that in colorectal cancer, CHGA is more predictive of early diagnosis than other biomarkers, such as KRAS and TP53 (Zhang et al., 2019). High expression of DOCK4 is closely related to invasive breast cancer and subsequent bone metastasis, making it a potentially useful biomarker to predict the risk of tumor bone metastasis (Westbrook et al., 2019). In addition, the role of biomarkers in lung cancer has also been reported. UCK2 may be a biomarker for early diagnosis and prognosis of lung cancer (Wu et al., 2019). In NSCLC, the level of serum IDH2 can be regarded as an effective biomarker for the diagnosis and prognosis (Li et al., 2018b), and LRP12 DNA methylation can be used as predictive biomarker for carboplatin resistance (Grasse et al., 2018).

As shown in Table 1, many of the 20 key genes have shown significant potential as biomarkers for CD8^+^ T cells. We will discuss several high confidence genes and try to show potential mechanisms of these genes in CD8^+^ T cells.

Pro-inflammatory protein S100A10, a member of the S100 protein family, is a crucial plasminogen receptor and is reported to be involved in the regulation of various intracellular processes such as cell cycle progression, transcription, and differentiation (Kwon et al., 2005). S100A10 is overexpressed in various cancers and plays a role in facilitating cell invasiveness by regulating pericellular proteolysis (Choi et al., 2003; Ji et al., 2004; Zhang et al., 2004). S100A10 is mainly expressed in regions with strong proliferation capacity (Petersson et al., 2009). Yang et al. observed that the reduction of availability of S100A10 had negative impact on the growth of tumor cells *in vitro* (Yang et al., 2011), suggesting the role of S100A10 in regulating cell proliferation. The current study by Katono et al. (2016) showed that S100A10 expression was significantly associated with high TNM stage, poor overall prognosis, and frequent vascular invasion. Moreover, several studies have shown that up-regulation of S100A11 is significantly associated with lymph node metastasis in patients with NSCLC (Tian et al., 2007; Yang et al., 2011; Katono et al., 2016).

CXCL13 (C-X-C motif chemokine ligand 13) is an antimicrobial peptide and CXC chemokine strongly expressed in the follicles of the spleen and lymph node. Recent studies recognized that the CXCR5–CXCL13 axis is involved in tumor dissemination to lymph nodes (Meijer et al., 2006; Singh et al., 2014). Analysis of serum CXCL13 levels in both subtypes of NSCLCs, squamous cell carcinoma (SCC) and adenocarcinoma (AC), showed that serum CXCL13 levels in ACs were higher than that in SCCs; this may be associated with patient prognosis (Singh et al., 2014). These findings of Singh et al. (2014) indicated that CXCR5 and CXCL13 may be useful as prognostic biomarkers for NSCLC. Smoke and air pollution are well known to be associated with lung cancer (Li et al., 2013; Berrandou et al., 2018), both of which contain polycyclic aromatic hydrocarbons (PAHs), a carcinogenic substance (Baxter et al., 2014). The experiments conducted by Wang et al. (2015) showed that CXCL13 levels of lung epithelial cells, of cancer cells, and of mice exposed to PAHs led to increased rates of lung cancer in mice, demonstrating that CXCL13 has an important role in PAH-induced lung cancers. Thommen et al. (2018) reported that CXCL13 plays an important role in the recruitment of lymphocytes to tertiary lymphoid structures.

S1PR1 is a G-protein-coupled receptor of the bioactive lipid sphingosine-1-phosphate (S1P) that is abundantly expressed in endothelial cells and blood (Cantalupo et al., 2017; Meissner, 2017) and plays a vital role in angiogenesis (Liu et al., 2000). Angiogenesis is a key process in the early stage of tumor progression and spread (Metodieva et al., 2011). Sarkisyan et al. (2014) suggested that S1PR1 signaling could delay tumor progression by enhancing or destabilizing integrity of neovasculature. S1PR signaling pathways are also reported to be involved in the oncogenesis of various cancers including NSCLC (Zhang et al., 2018b; Zhu et al., 2018). Apolipoprotein M (ApoM) is a sphingosine 1-phosphate (S1P) carrier, which is involved in regulating S1P (Duan et al., 2001; Sevvana et al., 2009). Overexpression of ApoM could promote proliferation, invasion, and tumor growth of NSCLC cell via upregulation of S1PR1 (Zhu et al., 2018).

Transgelin 2 (TAGLN2), an actin-binding protein, is overexpressed in various tumors and thought to be a tumor suppressor (Zhang et al., 2010; Jin et al., 2016; Han et al., 2017). Studies suggest that high levels of TAGLN2 in NSCLC cells were significantly associated with tumor development, neural invasion, and metastasis (Jin et al., 2016; Kim et al., 2018). Therefore, it has been considered a crucial diagnostic biomarker for early diagnosis and treatment guidance of NSCLC (Rho et al., 2009). Recent studies have focused on investigating microRNAs targeting TAGLN2 for tumor suppression (Nohata et al., 2011; Yoshino et al., 2011; Du et al., 2016).

### The Key Dysfunctional Pathway in CD8^+^ T Cells

As previously discussed, the most significantly enriched pathway of the 20-gene set is the hsa04062 chemokine signaling pathway. Three of these genes (CXCL13, RASGRP2, and PXN) are involved in this pathway.

Chemokines, a group of small molecular weight proteins, play an important role in cell migration, immune surveillance, and inflammation via binding to chemokine receptors on cell membranes (Raman et al., 2011). Numerous studies have shown the role of chemokine and chemokine receptors in the progression and metastasis of lung cancer (Cavallaro, 2013; Sarvaiya et al., 2013). The chemokine receptors, like CXCR4 and CCR7, are well studied (Mishan et al., 2016; Pozzobon et al., 2016). CXCR4 is the most commonly overexpressed and studied chemokine receptor in many different malignant tumors, including lung cancer (Balkwill, 2004). Differential expression of CXCR4 has been reported to be related to the metastatic potential of non-small cell lung cancer (NSCLC) (Phillips et al., 2003; Su et al., 2005). Wang et al. (2014) also revealed that the high-level CXCR4 expression was associated with brain-specific metastasis after complete resection of non-small cell lung cancer. In addition, CXCL12/CXCR4 axis is demonstrated to play a crucial role in migration and metastasis of NSCLC, and high expression of CXCL12/CXCR4 is related to poor prognosis in NSCLC (Suzuki et al., 2008). CCR7, a CC chemokine receptor, is mainly expressed on naive T cells, B cells, and mature dendritic cells (DCs) (Xu et al., 2012). Activation of CCR7 has been also proved to mediate the invasion and progression of NSCLC in most investigations (Cabioglu et al., 2007; Zhang L. et al., 2017). There was a correlation between tumor-infiltrating DC aggregation and apoptosis of NSCLC.

Besides CXCL13 as a chemokine, Paxillin (PXN) encodes a cytoskeletal protein, which contributes to actin-membrane attachment in the extracellular matrix. PXN is involved in signal transduction, which has been shown to be closely correlated with the oncogenesis and metastasis of NSCLC (Jagadeeswaran et al., 2008; Wu et al., 2010). Previous studies report that miR-137 suppressed cell migration and invasion by targeting PXN, therefore providing a potential therapy for NSCLC by targeting miRNA expression (Dacic et al., 2010; Bi et al., 2014). The expression of paxillin has also been observed as closely associated with the prognosis and the lymph node metastasis of NSCLC patients (Salgia et al., 1999; Zuo and Li, 2003; Wu et al., 2010). These studies strongly suggest the role of PXN in NSCLC, and thus, PXN is recommended as a potential target for NSCLC treatment.

## Conclusion

For more effective immunotherapy in the case of non-small cell lung cancers (NSCLC), we require further knowledge of T-cell biology. Therefore, we collected the published RNA-Seq gene expression data of 36 T-cell samples isolated from tumor and 32 adjacent uninvolved lung samples from a publicly available database. By analyzing them with a Monte Carlo feature selection method, we identified the discriminative genes between tumor T cells and adjacent uninvolved lung cells. In addition, we investigated the expression pattern of these key genes for CD8^+^ T cells of non-small cell lung cancer and their biological functions and pathways. However, tumors are commonly heterogeneous at the cellular level, and therefore, there are different proportions of CD8^+^ T-cell types (Wagner et al., 2019). This is currently an unresolved question.

## Data Availability Statement

We downloaded the gene expression profiles from Gene Expression Omnibus (GEO) under accession number GSE90728.

## Author Contributions

DM and TH designed the experiment. XT and XW performed the experiment and analyzed the data. XT and TH wrote the manuscript.

## Conflict of Interest

The authors declare that the research was conducted in the absence of any commercial or financial relationships that could be construed as a potential conflict of interest.

## Footnotes

1 https://cran.r-project.org/web/packages/e1071/
